# Strong Association between Two Polymorphisms on 15q25.1 and Lung Cancer Risk: A Meta-Analysis

**DOI:** 10.1371/journal.pone.0037970

**Published:** 2012-06-06

**Authors:** Mingliang Gu, Xiaoqun Dong, Xuezhi Zhang, Xumin Wang, Yue Qi, Jun Yu, Wenquan Niu

**Affiliations:** 1 Beijing Institute of Genomics, Chinese Academy of Sciences and Key Laboratory of Genome Science and Information, Chinese Academy of Sciences, Beijing, China; 2 Department of Biomedical and Pharmaceutical Sciences, College of Pharmacy, The University of Rhode Island, Kingston, Rhode Island, United States of America; 3 Clinical Laboratory of Biochemistry, Central Hospital of Shengli Oil Field, China Petrochemical Corporation, Dongying, Shandong, China; 4 Department of Epidemiology, Capital Medical University Affiliated Beijing Anzhen Hospital, Beijing Institute of Heart, Lung and Blood Vessel Diseases, Beijing, China; 5 State Key Laboratory of Medical Genomics at Ruijin Hospital, Shanghai Jiao Tong University School of Medicine, Shanghai, China; College of Pharmacy, University of Florida, United States of America

## Abstract

**Background:**

The association between polymorphisms on 15q25.1 and lung cancer has been widely evaluated; however, the studies have yielded contradictory results. We sought to investigate this inconsistency by performing a comprehensive meta-analysis on two polymorphisms (*CHRNA3* gene: rs1051730 and *AGPHD1* gene: rs8034191) on 15q25.1.

**Methods:**

Data were extracted from 15 and 14 studies on polymorphisms rs1051730 and rs8034191 involving 12301/14000 and 14075/12873 lung cancer cases/controls, respectively. The random-effects model was applied, addressing heterogeneity and publication bias.

**Results:**

The two polymorphisms followed Hardy-Weinberg equilibrium for all studies (*P*>0.05). For rs1051730-G/A, carriers of A allele had a 36% increased risk for lung cancer (95% confidence interval [CI]: 1.27–1.46; *P<*0.0005), without heterogeneity (*P* = 0.258) or publication bias (*P_Egger_* = 0.462). For rs8034191-T/C, the allelic contrast indicated that C allele conferred a 23% increased risk for lung cancer (95% CI: 1.08–1.4; *P* = 0.002), with significant heterogeneity (*P<*0.0005), without publication bias (*P_Egger_* = 0.682). Subgroup analyses suggested that the between-study heterogeneity was derived from ethnicity, study design, matched information, and lung cancer subtypes. For example, the association of polymorphisms rs1051730 and rs8034191 with lung cancer was heterogeneous between Caucasians (OR = 1.32 and 1.22; 95% CI: 1.25–1.44 and 1.05–1.42; *P*<0.0005 and 0.008, respectively) and East Asians (OR = 1.51 and 1.03; 95% CI: 0.76–3 and 0.47–2.27; *P* = 0.237 and 0.934, respectively) under the allelic model, and this association was relatively strengthened under the dominant model. There was no observable publication bias for both polymorphisms.

**Conclusions:**

Our findings demonstrated that *CHRNA3* gene rs1051730-A allele and *AGPHD1* gene rs8034191-T allele might be risk-conferring factors for the development of lung cancer in Caucasians, but not in East-Asians.

## Introduction

Lung cancer is the most common malignancy and the first-leading cause of cancer mortality, with an estimated 1.3 million new cases diagnosed annually in the world [1], [2]. The well-known risk factors for lung cancer include cigarette smoking and exposure to ionizing radiation (e.g., radon, medical imaging). Accumulating evidence has suggested that genetic factors may contribute to the variation in susceptibility to lung cancer. It is widely accepted that lung cancer is a complex multifactorial disease, attributed to the interaction of genetic factors with environmental factors [3], [4]. Despite intensive efforts devoted to investigating the genetic factors for lung cancer, the driving genes and genetic variants that determine the development of lung cancer are unclear.

The chromosome 15q25.1 region has been identified as a hotspot for lung cancer susceptibility by recent genome-wide association (GWA) studies [5], [6], [7], [8]. Results of genetic association studies for nicotine dependence, smoking behavior, and smoking-related diseases have converged to implicate the chromosome 15q25.1 region. The relationship between polymorphisms rs1051730 in *CHRNA3* gene and rs8034191 in the *AGPHD1* gene and lung cancer risk or related phenotypes has been widely investigated. As stated by McClellan and King, many if not most of the genetic polymorphisms that are reported to be associated with common disorders in GWA studies are factually spurious associations caused by subtle differences in ancestry between the populations being studied (known as “cryptic population stratification”) [9]. Moreover, based on the fact that individual studies with insufficient sample sizes lack sufficient statistical power to detect the common variants with tiny effects on lung carcinogenesis, the results are not reproducible. To derive a more precise estimation and investigate the inconsistency, we evaluated the effect of two polymorphisms rs1051730 and rs8034191 on the risk of lung cancer, addressing heterogeneity and publication bias.

## Methods

We performed this analysis in accordance with the guidelines of the Preferred Reporting Items for Systematic Reviews and Meta-analyses (PRISMA) statement [10] (see flowchart S1 and checklist S1).

### Search Strategy for Identification of Studies

We searched the PubMed and EMBASE databases for articles published before January 2012, using the Boolean combinations of subject terms (CHRNA3 OR AGPHD1 OR LOC123688) AND (lung cancer OR carcinoma OR neoplasm) AND (gene OR polymorphism OR allele OR genotype OR variant OR mutation). Articles were restricted to English-language and human studies. The full text of the retrieved articles was scrutinized to decide whether information on the topic of interest was included. Reference lists of these retrieved articles and systematic reviews were also checked for citations of articles not initially identified. For articles involving more than one geographic or ethnic heterogeneous group, each group was treated separately. When genotype frequency was not reported, we contacted the authors to obtain the relevant information.

### Inclusion/Exclusion Criteria

Articles were included in this meta-analysis if they 1) examined the hypothesis that *CHRNA3* gene rs1051730 polymorphism and/or *AGPHD1* gene rs8034191 polymorphism were associated with lung cancer risk; 2) followed a nested case-control or case-control or cross-sectional study design; and 3) provided sufficient information on genotype/allele counts between cases and controls to estimate the odds ratio (OR) and the corresponding 95% confidence interval (95% CI). The relatively complete and recent results were extracted when there were multiple articles involving the same population.

### Extracted Information

The following information was extracted independently and entered into separate databases by two authors (MG and WN) from each qualified study: first author's last name, publication date, population ethnicity, study design, baseline characteristics of the study population including age, ethnicity, sex, smoking status, and the genotype counts in cases and controls. Any encountered discrepancy was adjudicated by a discussion until a consensus was reached.

### Quality Score Assessment

The study quality was assessed by using a quality assessment score developed for genetic association studies by Thakkinstian et al [11]. Total scores ranged from 0 (worst) to 12 (best). The criteria for quality assessment of the genetic association between two studied polymorphisms and lung cancer are described in Table S1.

### Statistical Analysis

Data management and statistical analyses were conducted using STATA software (StataCorp, Texas, USA, version 11.0 for Windows). Deviation from Hardy-Weinberg equilibrium was tested by χ^2^ or Fisher's exact test in control groups. Irrespective of between-study heterogeneity, a random-effects model using the DerSimonian and Laird method was implemented to bring the individual effect-size estimates together, and the estimate of heterogeneity was taken from the Mantel-Haenszel model [12]. Unadjusted OR and 95% CI were used to compare allelic and dominant contrast between cases and controls.

Between-study heterogeneity was assessed by the inconsistency index *I* ^2^ statistic (ranging from 0 to 100%), which was documented for the percentage of the observed between-study variability due to heterogeneity rather than by chance, with higher values suggesting the existence of heterogeneity [13], [14]. In the case of between-study heterogeneity, we examined the study characteristics that could stratify the studies into subgroups with homogeneous effects. To estimate the extent to which one or more covariates explained the heterogeneity, we employed meta-regression, as an extension of random-effects meta-analysis.

Cumulative meta-analysis was conducted to identify the influence of the first published study on the subsequent publications, and the evolution of the combined estimates over time according to the ascending date of publication. To identify potentially influential studies, we performed influential analysis (also known as sensitivity analysis) by removing an individual study each time to check whether any of these estimates biased the overall estimate.

The funnel plot and Egger's test were applied to assess publication bias [15]. Egger's test can detect funnel plot asymmetry by determining whether the intercept deviates significantly from zero in a regression of the standardized effect estimates against their precision. Trim and fill method was also used to estimate the number and outcomes of potentially missing studies resulting from publication bias. A probability <0.05 was considered significant except for the *I* ^2^ and Egger's statistic, for which a significance level was defined as <0.1.

## Results

### Search of Studies

**Figure 1:**
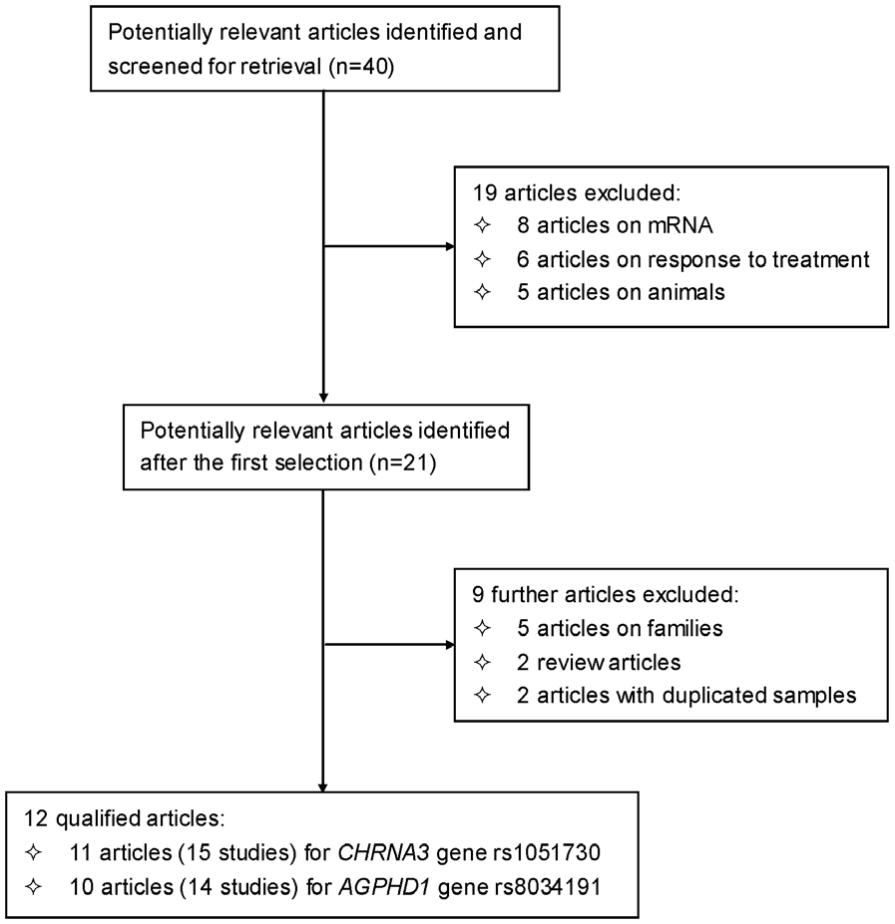
Flow diagram of search strategy and study selection.

Based on our search strategy, the primary screening produced 40 potentially relevant articles, of which 12 met the inclusion criteria in an attempt to evaluate the association of *CHRNA3* gene rs1051730 and/or *AGPHD1* gene rs8034191 polymorphisms with lung cancer risk [5], [16], [17], [18], [19], [20], [21], [22], [23], [24], [25], [26]. A flow diagram schematized the process of selecting and excluding articles with specific reasons (Figure 1). The 12 qualified articles were published between 2008 and 2011 involving 16 studies with 9 in Caucasians, 4 in East Asians, 2 in African-Americans, and 1 in mixed (Caucasian, African-American and Hispanic) populations. The quality score of studies ranged from 7 to 10 (mean: 8.5) out of a maximal score of 12. In detail, there were 11 (15) and 10 (14) articles (studies) for rs1051730 and rs8034191 polymorphisms involving 12301/14000 and 14075/12873 lung cancer cases/controls, respectively.

### Study Characteristics

The baseline characteristics of all qualified studies are summarized in Table 1. Genotype distributions of two polymorphisms were in Hardy-Weinberg equilibrium for all studies (*P*>0.05). Ten of 16 qualified studies were matched on age or sex or smoking status between cases and controls [16], [18], [19], [21], [22], [24], [25], [26]. Five studies were hospital-based [16], [19], [23], [26], and the rest were population-based. Three studies involved non-small-cell lung cancer as an end point, and one study involved squamous cell lung carcinoma as an end point. The frequencies of *CHRNA3* gene rs1051730-A allele ranged widely between Caucasians and East Asians with African-Americans in between. For example in control groups, the rs1051730-A allele ranged from 29.45% to 37.14% in Caucasians, from 1.39% to 3.3% in East Asians, and from 16.15% to 19.85% in African-Americans. The observation was similar for *AGPHD1* gene rs8034191-C allele, with frequencies ranging from 23.04% to 39.47% in Caucasian controls, from 1.82% to 3.72% in East Asian controls, and from 16.15% to 31.44 in African-American controls.

**Table 1:** The baseline characteristics of all qualified studies in this meta-analysis.

First author	Disease type	Match	Ethnicity	Design	Age, years		Gender (Males, %)	
					Cases	Controls	Cases	Controls
Zienolddiny S et al.	NSCLC	age, sex, smoking	Caucasian	population	60	60	76.99	76.14
Schwartz AG et al.	NSCLC	age, sex, race	Caucasian	population	NA	NA	NA	NA
Schwartz AG et al.	NSCLC	age, sex, race	African-American	population	NA	NA	NA	NA
Liu P et al.	lung cancer	NA	Caucasian	population	61.3	75.6	42.27	58.9
Liu P et al.	lung cancer	NA	Caucasian	population	64.6	57.1	56.1	54.9
Liu P et al.	lung cancer	NA	Caucasian	population	64.5	60.7	49.9	35.3
Spitz MR et al.	lung cancer	age, sex, race	Mixed*	population	NA	NA	NA	NA
Amos CI et al.	lung cancer	NA	Caucasian	hospital	62.1	61.1	57	56.6
Girard N et al.	lung cancer	age	Caucasian	population	NA	NA	NA	NA
Girard N et al.	lung cancer	age	Japanese	population	NA	NA	NA	NA
Broderick P et al.	lung cancer	NA	Caucasian	population	NA	NA	NA	NA
Shiraishi K et al.	lung cancer	NA	Japanese	hospital	60	50	73	60
Kohno T et al.	LSCC	sex, smoking	Japanese	hospital	62.7	62.5	90	57
Sakoda LC et al.	lung cancer	age, sex, smoking	Caucasian	population	NA	NA	67.3	66.6
Amos CI et al.	lung cancer	age, sex, race	African-American	hospital	62.4	55.7	54.82	41.24
Wu C et al.	lung cancer	age, sex	Chinese	hospital	NA	NA	69.3	31.1

Author	Smoking status (%)		Quality score	rs1051730-A (%)		rs8034191-C (%)	
	Cases	Controls		Cases	Controls	Cases	Controls
Zienolddiny S et al.	current/former: 79.7/20.3	80.9/19.1	9	42.61	36.12	41.48	36.44
Schwartz AG et al.	current/former: 17.6/82.4	52.9/47.1	9	41.19	37.02	41.19	37.02
Schwartz AG et al.	current/former: 10.2/89.8	45.1/54.9	9	19.31	16.15	19.31	16.15
Liu P et al.	current/never: 86.6/13.4	6.4/93.6	7	40.72	29.45	43.56	29.22
Liu P et al.	current/former/never: 43.9/56.1/0	55.2/44.8/0	7	39.71	33.41	39.65	33.47
Liu P et al.	current/former/never: 27.9/65.8/6.25	23.0/40.1/36.9	7	39.29	31.6	39.99	31.71
Spitz MR et al.	NA	NA	8	39.62	33.47	NA	NA
Amos CI et al.	current/former/never: 47.8/52.3/0	42.2/57.8/0	10	39.77	33.47	39.81	33.69
Girard N et al.	NA	NA	8	34.57	37.14	35.64	39.47
Girard N et al.	NA	NA	8	1.63	1.42	1.63	2.63
Broderick P et al.	NA	NA	8	NA	NA	18.78	23.04
Shiraishi K et al.	current/never: 79/21	39/61	9	3.32	1.39	3.52	1.82
Kohno T et al.	current/never: 97/3	45/55	8	3.34	1.54	NA	NA
Sakoda LC et al.	current/former: 72.4/27.6	72.4/27.6	10	40.74	34.24	40.68	34.2
Amos CI et al.	current/former/never: 48.82/40.04/11.13	37.37/36.08/26.55	9	33.4	19.85	41.97	31.44
Wu C et al.	current/former/never: 52.5/8.7/38.8	27.7/8.8/63.5	10	2.74	3.3	2.81	3.72

*Abbreviations:* NSCLC, non-small-cell lung cancer; SLCC, lung squamous cell carcinoma; NA, not available.

* Mixed ethnicity included Caucasian, African-American and Hispanic.

### Overall Analysis

Due to the sparseness of the mutant alleles of both studied polymorphisms in East Asians and to maximize the statistical power to detect an association, we considered the risk effect of two polymorphisms under both allelic and dominant models.

The overall comparison of *CHRNA3* gene rs1051730-A allele yielded a remarkably increased risk for lung cancer (OR = 1.33; 95% CI: 1.24–1.44; *P<*0.0005) relative to the rs1051730-G allele; however, there was moderate evidence of between-study heterogeneity (*I* ^2^ = 57.8%; *P* = 0.003). The risk magnitude was slightly potentiated under the dominant model (OR = 1.36; 95% CI: 1.27–1.46; *P<*0.0005), but heterogeneity was absent (*I* ^2^ = 18.4%; *P* = 0.258) (Table 2). Likewise for *AGPHD1* gene rs8034191-T/C polymorphism, as compared with T allele, the C allele conferred a significant 23% increased risk for lung cancer (95% CI: 1.08–1.4; *P* = 0.002) with heterogeneity (*I* ^2^ = 87.2%; *P*<0.0005). The risk magnitude was slightly weakened under the dominant model (OR = 1.2; 95% CI: 1.02–1.42; *P* = 0.03; *I* ^2^ = 85.2%; *P*<0.0005) (Table 3).

**Table 2:** Overall and subgroup analyses of *CHRNA3* gene rs1051730 polymorphism with the odds of developing lung cancer under allelic and dominant models.

Overall & subgroups	Studies (cases/controls), n(n/n)	Allelic model: A vs. G		Dominant model: AA+AG vs. GG	
		OR; 95% CI; P	*I* ^2^ (%); *P*_χ2_; *P*_Egger_	OR; 95% CI; P	*I* ^2^ (%); *P*_χ2_; *P*_Egger_
**Total studies**	15 (12301/14000)	1.33; 1.24–1.44; <0.0005	57.8; 0.003; 0.742	1.36; 1.27–1.46; <0.0005	18.4; 0.258; 0.462
***Ethnicity***					
**Caucasian**	8 (6955/9001)	1.32; 1.25–1.4; <0.0005	24.0; 0.238; 0.342	1.39; 1.31–1.49; <0.0005	0.0; 0.831; 0.357
**East Asian**	4 (2898/2657)	1.51; 0.76–3.0; 0.237	82.1; 0.001; 0.623	1.22; 0.59–2.52; 0.592	66.9; 0.049; 0.517
**African-American**	2 (594/615)	1.57; 0.97–2.54; 0.064	81.8; 0.019; NA	1.38; 1.02–1.87; 0.034	NA
**Mixed**	1 (1854/1727)	1.3; 1.18–1.44; <0.0005	NA	1.35; 1.18–1.55; <0.0005	NA
***Matched***					
**Yes** *	10 (5826/6877)	1.27; 1.13–1.42; <0.0005	57.1; 0.013; 0.891	1.31; 1.15–1.48; <0.0005	38.3; 0.113; 0.637
**NA**	5 (6475/7123)	1.41; 1.27–1.55; <0.0005	58.1; 0.049; 0.675	1.4; 1.3–1.51; <0.0005	0.0; 0.853; 0.827
***Study design***					
**Hospital**	5 (4162/3741)	1.56; 1.1–2.21; 0.012	82.7; <0.0005; 0.493	1.24; 0.79–1.96; 0.348	78.9; 0.009; 0.971
**Population**	10 (8139/10259)	1.32; 1.26–1.39; <0.0005	6.4; 0.383; 0.312	1.39; 1.31–1.48; <0.0005	0.0; 0.931; 0.34
***Type of lung cancer***					
**Mixed**	11 (10676/12021)	1.35; 1.24–1.48; <0.0005	65.5; 0.001; 0.838	1.34; 1.23–1.46; <0.0005	34.6; 0.141; 0.133
**NSCLC**	3 (1251/1655)	1.24; 1.1–1.38; <0.0005	0.0; 0.76; 0.619	1.38; 1.18–1.61; <0.0005	0.0; 0.683; 0.372
**LSCC**	1 (374/324)	2.21; 1.05–4.63; 0.036	NA	2.25; 1.06–4.76; 0.034	NA

*Abbreviations:* NA, not available; OR, odds ratio; 95% CI, 95% confidence interval; NSCLC, non-small-cell lung cancer; LSCC, lung squamous cell carcinoma.

* Matched on age or sex or smoking.

**Table 3:** Overall and subgroup analyses of *AGPHD1* gene rs8034191 polymorphism with the odds of developing lung cancer under allelic and dominant models.

Overall & subgroups	Studies (cases/controls), n(n/n)	Allelic model: C vs. T		Dominant model: CC+CT vs. TT	
		OR; 95% CI; P	*I* ^2^ (%); *P*_χ2_; *P*_Egger_	OR; 95% CI; P	*I* ^2^ (%); *P*_χ2_; *P*_Egger_
**Total studies**	14 (14075/12837)	1.23; 1.08–1.4; 0.002	87.2; <0.0005; 0.682	1.2; 1.02–1.42; 0.03	85.2; <0.0005; 0.536
***Ethnicity***					
**Caucasian**	9 (10968/9897)	1.22; 1.05–1.42; 0.008	90.3; <0.0005; 0.68	1.26; 1.06–1.51; 0.011	87.1; <0.0005; 0.921
**East Asian**	3 (2513/2325)	1.03; 0.47–2.27; 0.934	86.2; 0.001; NA	0.72; 0.52–0.99; 0.043	0.0; 0.762; NA
**African-American**	2 (594/615)	1.39; 1.1–1.76; 0.006	32.6; 0.223; NA	1.38; 1.02–1.87; 0.034	NA
***Matched***					
**Yes** *	8 (3588/4807)	1.17; 1.02–1.35; 0.029	60.1; 0.014; 0.139	1.17; 0.96–1.43; 0.127	62.6; 0.014; 0.123
**NA**	6 (10487/8030)	1.34; 1.07–1.67; <0.0005	94.0; <0.0005; 0.217	1.27; 0.96–1.66; 0.09	93.1; <0.0005; 0.988
***Study design***					
**Hospital**	4 (3777/3409)	1.31; 0.96–1.79; 0.092	82.1; 0.001; 0.974	0.99; 0.56–1.76; 0.982	89.3; 0.002; NA
**Population**	10 (10289/9428)	1.2; 1.03–1.41; 0.024	89.3; <0.0005; 0.588	1.25; 1.03–1.51; 0.021	85.9; <0.0005; 0.79
***Lung cancer type***					
**Mixed**	11 (12824/11183)	1.23; 1.04–1.45; 0.014	90.1; <0.0005; 0.73	1.15; 0.93–1.42; 0.211	89.0; <0.0005; 0.459
**NSCLC**	3 (1251/1654)	1.21; 1.08–1.36; 0.001	0.0; 0.944; 0.27	1.35; 1.16–1.58; <0.0005	0.0; 0.859; 0.207

*Abbreviations:* NA, not available; OR, odds ratio; 95% CI, 95% confidence interval; NSCLC, non-small-cell lung cancer.

* Matched on age or sex or smoking.

**Figure 2:**
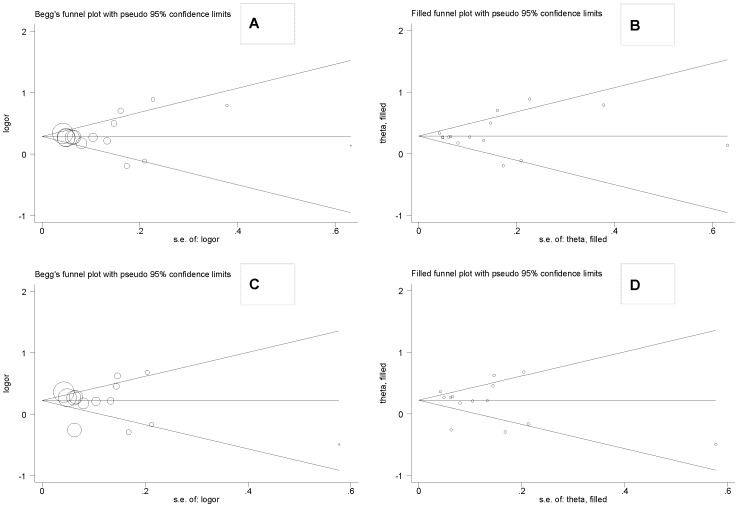
Funnel and filled funnel plots for studies investigating the effect of *CHRNA3* gene polymorphism rs1051730 (A and B) and *AGPHD1* gene polymorphism rs8034191 (C and D) on the risk of lung cancer.

**Figure 3:**
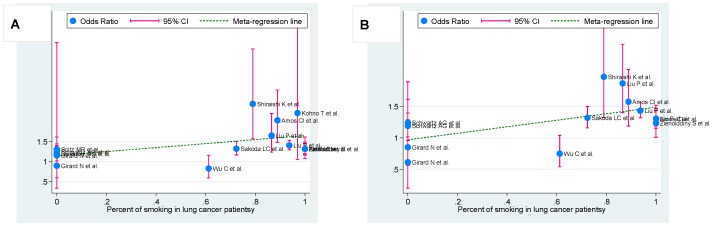
Meta-regression of smoking percent in lung cancer patients on in-allele risk estimates of *CHRNA3* gene rs1051730 (A) and *AGPHD1* gene rs8034191 (B) polymorphisms for occurrence of lung cancer. For each study, OR is shown by the middle of the blue solid circle whose upper and lower extremes represent the corresponding 95% CI. OR values were calculated for the current smokers against nonsmokers (including former smokers) when available or ex-smokers against never-smokers otherwise. The green dotted line is plotted by fitting OR and smoking percent in cases for the included studies.

For both polymorphisms, as reflected by the visual funnel plot inspection (Figure 2-A and 2-C) and Egger's regression asymmetry statistic, there was low probability of publication bias (*P* = 0.742 and 0.682 for rs1051730 and rs8034191, respectively). Further evidence of selective publication suggested that there were no missing studies required to make the funnel plot symmetrical for both polymorphisms (Figure 3-B and 3-D).

### Cumulative and Influential Analyses

In the cumulative meta-analysis, across all genetic models we found no evidence suggesting that the first published study that reported a potentially significant result then triggered subsequent publication replication. The influential analysis showed that no single study influenced the overall results significantly for both polymorphisms (data not shown).

### Subgroup Analysis

In view of significant heterogeneity and to seek for its potential sources, we performed a panel of subgroup analyses on ethnicity, matched information, study design, and disease type.

Grouping studies by descent of populations indicated that the odds of developing lung cancer was significantly augmented in African-Americans for both polymorphisms, and was non-significant or remarkably lowered in East Asians for *CHRNA3* gene rs1051730 polymorphism (allelic model: OR = 1.51; 95% CI: 0.76–3.0; *P* = 0.237; and dominant model: OR = 1.22; 95% CI: 0.59–2,52; *P* = 0.592) (Table 2), and *AGPHD1* gene rs8034191 polymorphism (allelic model: OR = 1.03; 95% CI: 0.47–2.27; *P* = 0.934; and dominant model: OR = 0.72; 95% CI: 0.52–0.99; *P* = 0.043) (Table 3). In contrast, there were no material changes in risk estimates in Caucasians for both polymorphisms. Upon stratification by the matched information on age or gender or smoking status between cases and controls, the risk estimates were relatively weakened in matched studies for both polymorphisms under both allelic and dominant models (Tables 2 and 3), and the quality of heterogeneity was not improved.

In subgroup analysis by study design, association of both studied polymorphisms with lung cancer was potentiated in hospital-based studies under allelic model (rs1051730: OR = 1.56, 95% CI: 1.1–2.21, *P* = 0.012; and rs8034191: OR = 1.31, 95% CI: 0.96–1.79, *P* = 0.092), whereas under dominant model, this association was potentiated in population-based studies (rs1051730: OR = 1.39, 95% CI: 1.31–1.48, *P*<0.0005; and rs8034191: OR = 1.25, 95% CI: 1.03–1.51, *P* = 0.021). Restricting analysis to the non-small-cell lung cancer observed no evidence of heterogeneity, and found that the risk magnitude was significant but relatively weakened under allelic model (rs1051730: OR = 1.24, 95% CI: 1.1–1.38, *P*<0.0005; and rs8034191: OR = 1.21, 95% CI: 1.08–1.36, *P* = 0.001), whereas this magnitude was significantly strengthened under dominant model (rs1051730: OR = 1.38, 95% CI: 1.18–1.61, *P*<0.0005; and rs8034191: OR = 1.35, 95% CI: 1.16–1.58, *P*<0.0005).

### Meta-regression analysis

To identify other sources of heterogeneity, we undertook meta-regression analysis of age (mean or median value), sex (male percent), and smoking rate (percentage of current and former smokers). Among these variables, the association of *CHRNA3* gene rs1051730 (correlation coefficient: 0.48, *P* = 0.069) and *AGPHD1* gene rs8034191 (correlation coefficient: 0.57, *P* = 0.043) polymorphisms with lung cancer risk was observed in cases with a high smoking rate under the allelic model (Figure 3).

## Discussion

Via a comprehensive meta-analysis, we evaluated the association of two common polymorphisms on 15q25.1 with the risk of lung cancer. Overall results demonstrated that *CHRNA3* gene rs1051730-A allele and *AGPHD1* gene rs8034191-T allele might be risk-conferring factors for the development of lung cancer in Caucasians, but not in East-Asians. Although potential sources of heterogeneity could not be easily eliminated, the present study, to our knowledge, is the first meta-analysis to date dealing with the association of these two polymorphisms with lung cancer susceptibility.

We identified ethnicity as a potential source of between-study heterogeneity by subgroup analysis. Genetic heterogeneity is inevitable in disease identification strategy [27]. We found that the association of rs1051730 and rs8034191 polymorphisms with lung cancer risk was heterogeneous between Caucasians and East Asians. The significance was observed only in the former, which consisted with the results of GWA studies from western populations. We also have noticed remarkable differences in *CHRNA3* gene rs1051730-A allele and *AGPHD1* gene rs8034191-C allele between Caucasians and East Asians, making it very difficult to detect the weak association in Asians unless examining a very large population. This suggests that different genetic backgrounds may cause this discrepancy or that different populations may have different linkage disequilibrium patterns. The studied polymorphisms may be in linkage with another causal variant in one ethnic population but not in another [28]. For example, the rs1051730 polymorphism is in complete linkage disequilibrium with the potentially pathogenic allele of rs16969968 (D398N) in the *CHRNA5* gene [17], [29]. We therefore speculate that polymorphism rs1051730 may have a pleiotropic effect on the etiology of lung carcinogenesis across different ethnic groups. In view of the divergent genetic backgrounds, it is necessary to construct a database of polymorphisms related to lung cancer in each ethnic/racial group.

Besides the disturbing influence of ethnicity on overall estimate, any estimate should be treated with caution when studies were stratified by study design. In this meta-analysis, for both polymorphisms, the risk estimates in hospital-based studies were stronger than that in population-based studies. Besides the relatively small sample size, drawbacks of hospital-based studies should not be disregarded, as population stratification remains an important issue [30]. Two studies had recruited subjects from only one hospital, and thus there might be a narrow socioeconomic profile for both cases and controls. In addition, poor comparability between cases and controls in hospital-based studies might exert a confounding effect on the true association in light of a regional specialty for the disease and the differential hospitalization rates between cases and controls [31]. In contrast, subjects drawn from the community or the general population might be more representative of the population, making the results from population-based studies more convincing. Considering the wider confidence intervals of estimates, more studies are required to quantify the effect size reliably.

Furthermore, our meta-regression analysis found an association of two studied polymorphisms with lung cancer risk in patients with a higher smoking rate. We defined smoking rate based on the percentage of current and former smokers if available. This definition is unlikely to undermine our observation since the exclusion of ever-smoking might lead to an underestimation of the risk for lung cancer. Moreover, our data on smoking and other confounders were extracted from recent publications (after the year 2008) from professional cancer journals as reflected by the high quality score. Additionally, smoking is by far the major contributor to lung cancer, accounting for about 90% of the lung cancer incidence [32]. Previous studies demonstrated that polymorphisms in the *CHRNA3* gene were associated with an increased risk of smoking initiation, indicating a potential genotype-phenotype interaction [33].

The strengths of this study include the relatively large sample size, no deviation from Hardy-Weinberg equilibrium, and the high quality of the qualified studies. However, our current study should be interpreted with several technical limitations in mind. Firstly, most of the studies in this meta-analysis were case-control studies, which are susceptible to selection bias by including only nonfatal cases. Secondly, because only published studies in English were retrieved and the “grey” literature (articles in languages other than English) was not included, publication bias might be possible, even though our funnel plots and statistical tests did not show it. However, asymmetry in the funnel plot, being either visually interpreted or statistically tested, may result from an essential difference between the small and larger studies that arises from inherent between-study heterogeneity [34]. Because currently we have no golden standard to compare the results of funnel plot tests [34], Egger's test and the usual funnel plot have been challenged. We cannot completely rule out a low probability that small negative studies are missing from the plot. Nevertheless, the trim and fill method suggested no missing studies required to make the funnel plot symmetrical for both polymorphisms. Thirdly, the single locus–based nature of meta-analysis precluded the possibility of gene-gene and gene-environment interactions, as well as haplotype-based effects, suggesting that additional studies assessing these aspects are necessary. Fourthly, we focused only on two polymorphisms on 15q25.1 and did not consider other candidate genes or polymorphisms. It is likely that the studied polymorphisms by itself make a minor contribution to risk prediction in lung cancer patients, but whether the two polymorphisms when integrated with other risk factors will enhance the prediction requires further investigation.

Taken together, we have expanded previously individual studies by providing the convincing evidence that *CHRNA3* gene rs1051730-A allele and *AGPHD1* gene rs8034191-T allele might be risk-conferring factors for the development of lung cancer in Caucasians, but not in East-Asians. We have strengthened the previous findings on the association of high smoking rate with increased lung cancer risk. Further studies should investigate the markers on and adjacent to 15q25.1 to clarify whether the present association is causal or due to linkage disequilibrium.

## Supporting Information

Table S1 Criteria for quality assessment of genetic association of *CHRNA3* gene rs1051730 polymorphism and *AGPHD1* gene rs8034191 polymorphism with lung cancer.
(DOC)

Flowchart S1 PRISMA flowchart.
(PDF)

Checklist S1 PRISMA checklist.
(DOC)
