# Supplementary material for: Strong Association between Two Polymorphisms on 15q25.1 and Lung Cancer Risk: A Meta-Analysis
Source: PLoS One. 2012 Jun 6;7(6):e37970. doi: 10.1371/journal.pone.0037970 (PMC3368941; doi:10.1371/journal.pone.0037970)
Supplement: Table S1 — Criteria for quality assessment of genetic association of CHRNA3 gene rs1051730 polymorphism and AGPHD1 gene rs8034191 polymorphism with lung cancer. (DOC) [file pone.0037970.s001.doc]

**TABLE S1. Criteria for quality assessment of genetic association of *CHRNA3* gene rs1051730 polymorphism and *AGPHD1* gene rs8034191 polymorphism with lung cancer**

| **Criteria** | **Quality score** |
| --- | --- |
| ***Representativeness of case-patients*** | |
| 1. Consecutively/randomly selected from case population with clearly defined random frame | 2 |
| 1. Consecutive/randomly selected from case population without clearly defined random frame or with extensive inclusion criteria | 1 |
| 1. Method of selection not described | 0 |
| ***Representativeness of controls*** | |
| 1. Controls were consecutive/randomly drawn from the same area (ward/community) as cases with the same criteria | 2 |
| 1. Controls were consecutive/randomly drawn from a different area than were cases | 1 |
| 1. Not described | 0 |
| ***Ascertainment of lung cancer patients*** | |
| 1. Clearly described objective criteria for diagnosis of lung cancer | 1 |
| 1. Not described | 0 |
| ***Ascertainment of controls*** | |
| 1. Clinical examinations were performed on controls to prove that controls did not have lung cancer | 2 |
| 1. Article merely stated that controls were subjects who did not have lung cancer; no proof provided | 1 |
| 1. Not described | 0 |
| ***Ascertainment of genotyping examination*** | |
| 1. Genotyping performed under “blind” conditions | 1 |
| 1. Unblinded or not mentioned | 0 |
| ***Test for Hardy-Weinberg equilibrium*** | |
| 1. Hardy-Weinberg equilibrium in control group | 2 |
| 1. Hardy-Weinberg disequilibrium in control group | 1 |
| 1. Hardy-Weinberg equilibrium not checked | 0 |
| ***Association assessment*** | |
| 1. Assessed association between genotypes and lung cancer with appropriate statistics and adjusting for confounders | 2 |
| 1. Assessed association between genotypes and lung cancer with appropriate statistic without adjusting for confounders | 1 |
| 1. Inappropriate statistics used | 0 |
