# Supplementary figures and images for: Strong Association between Two Polymorphisms on 15q25.1 and Lung Cancer Risk: A Meta-Analysis
Source: PLoS One. 2012 Jun 6;7(6):e37970. doi: 10.1371/journal.pone.0037970 (PMC3368941; doi:10.1371/journal.pone.0037970)

## PRISMA flowchart

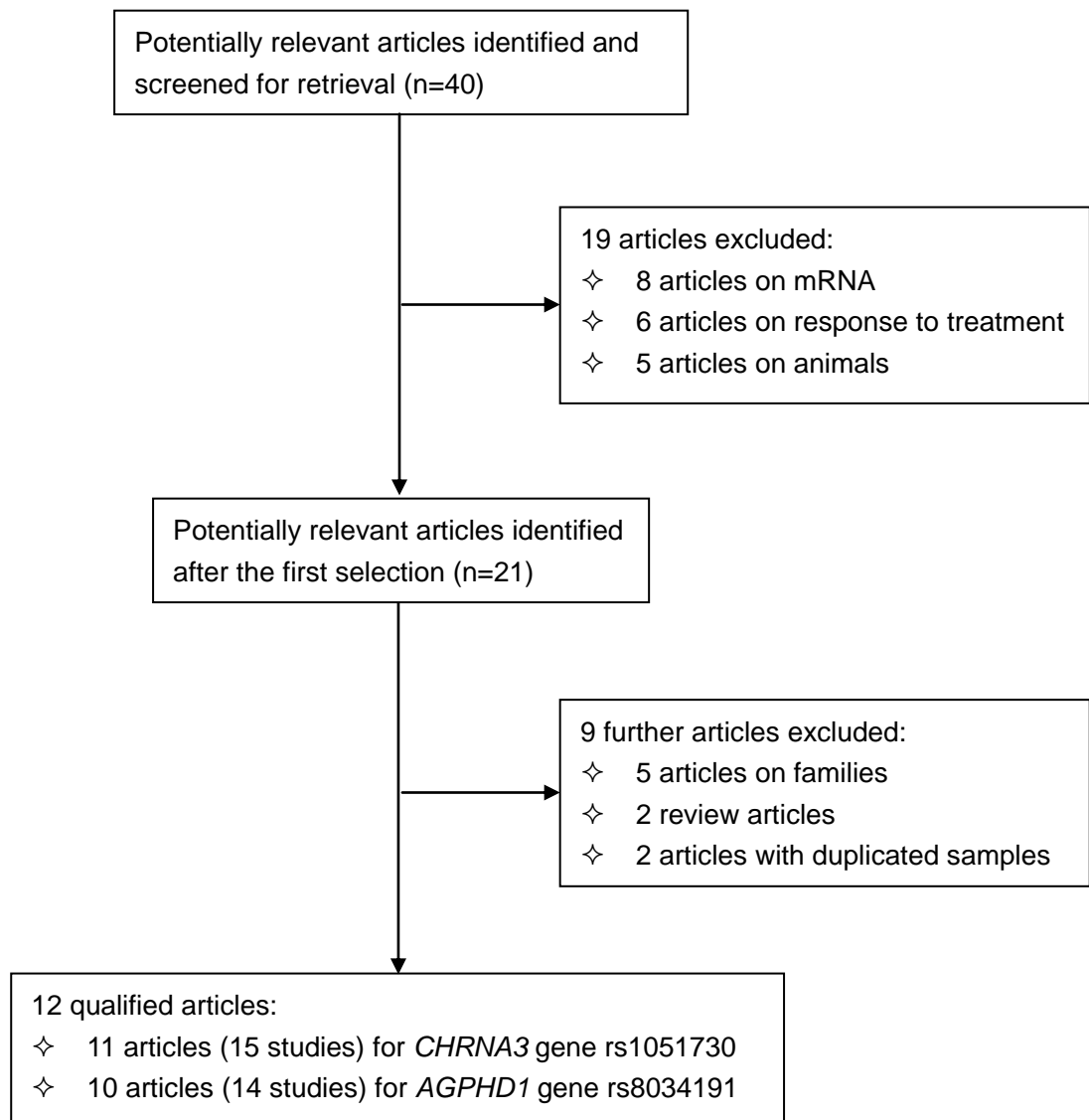

Supplement: Flowchart S1 — PRISMA flowchart. (PDF) [file pone.0037970.s002.pdf]
